# Sharing experiences: towards an evidence based model of dengue surveillance and outbreak response in Latin America and Asia

**DOI:** 10.1186/1471-2458-13-607

**Published:** 2013-06-24

**Authors:** Shiraz Badurdeen, David Benitez Valladares, Jeremy Farrar, Ernesto Gozzer, Axel Kroeger, Novia Kuswara, Silvia Runge Ranzinger, Hien Tran Tinh, Priscila Leite, Yodi Mahendradhata, Ronald Skewes, Ayesha Verrall

**Affiliations:** 1 Department of Paediatrics, University of Oxford, Oxford, UK; 2 Liverpool School of Tropical Medicine, Liverpool, UK; 3 Tropical Medicine, Oxford University Clinical Research Unit in Viet Nam, Oxford, UK; 4 Asociado FASPA/UPCH, Av. Honorio Delgado 430 San Martín de Porres, Lima, Perú; 5 HO/TDR, Geneva, Switzerland and School of Tropical Medicine, Liverpool, UK; 6 Ministry of Health Kuala Lumpur, Kuala Lumpur, Malaysia; 7 Public Health Specialist, Stuttgart, Germany; 8 Clinical Research, Centre for Tropical Medicine Oxford University Clinical Research Unit Vietnam (OUCRU) Wellcome Trust Major Overseas Programme (MOP), Ho Chi Minh City, Vietnam; 9 National Dengue Control Ministry of Health of Brazil Esplanada dos Ministérios, Ed sede, Bloco G, Sala 148, Brasilia/DF 70058-900, Brazil; 10 Research & Collaboration, Center for Tropical Medicine, Faculty of Medicine, Gadjah Mada University, Yogyakarta, Indonesia; 11 Ministerio de Salud República Dominicana, Santo Domingo, Dominican Republic; 12 Department of Medicine, National University Singapore, 1E Kent Ridge Road, NUHS Tower Block Level 11, Singapore 119228, Singapore

**Keywords:** Dengue outbreaks, Dengue epidemics, Outbreak response, Outbreak detection

## Abstract

**Background:**

The increasing frequency and intensity of dengue outbreaks in endemic and non-endemic countries requires a rational, evidence based response. To this end, we aimed to collate the experiences of a number of affected countries, identify strengths and limitations in dengue surveillance, outbreak preparedness, detection and response and contribute towards the development of a model contingency plan adaptable to country needs.

**Methods:**

The study was undertaken in five Latin American (Brazil, Colombia, Dominican Republic, Mexico, Peru) and five in Asian countries (Indonesia, Malaysia, Maldives, Sri Lanka, Vietnam). A mixed-methods approach was used which included document analysis, key informant interviews, focus-group discussions, secondary data analysis and consensus building by an international dengue expert meeting organised by the World Health Organization, Special Program for Research and Training in Tropical Diseases (WHO-TDR).

**Results:**

Country information on dengue is based on compulsory notification and reporting (“passive surveillance”), with laboratory confirmation (in all participating Latin American countries and some Asian countries) or by using a clinical syndromic definition. Seven countries additionally had sentinel sites with active dengue reporting, some also had virological surveillance. Six had agreed a formal definition of a dengue outbreak separate to seasonal variation in case numbers. Countries collected data on a range of warning signs that may identify outbreaks early, but none had developed a systematic approach to identifying and responding to the early stages of an outbreak. Outbreak response plans varied in quality, particularly regarding the early response. The surge capacity of hospitals with recent dengue outbreaks varied; those that could mobilise additional staff, beds, laboratory support and resources coped best in comparison to those improvising a coping strategy during the outbreak. Hospital outbreak management plans were present in 9/22 participating hospitals in Latin-America and 8/20 participating hospitals in Asia.

**Conclusions:**

Considerable variation between countries was observed with regard to surveillance, outbreak detection, and response. Through discussion at the expert meeting, suggestions were made for the development of a more standardised approach in the form of a model contingency plan, with agreed outbreak definitions and country-specific risk assessment schemes to initiate early response activities according to the outbreak phase. This would also allow greater cross-country sharing of ideas.

## Background

Dengue is the most rapidly expanding arboviral disease and dengue outbreaks exert a huge burden on populations, health systems and economies in most tropical countries [1]. Dengue-endemic countries and also a number of countries with low-level or no transmission are threatened by outbreaks that are detected at a late stage and where the response mechanisms are often inadequate. Here, the term “outbreak” (used synonymously with “epidemic”) is defined as a “sudden unexpected increase of cases” or as ‘the occurrence in a community or region of cases of an illness clearly in excess of expectancy’ [2]. Such a “sudden and unexpected increase” (outbreak) is different from the seasonal peak, which is an “expected increase in cases” that usually occurs at the end or after the wet season.

Early detection of outbreaks poses a challenge, since no universally accepted operational definition of an outbreak exists and methods for distinguishing between seasonal fluctuations and true outbreaks are not generally applied. Candidate indicators for predicting a dengue outbreak, or for early outbreak detection through “syndromic surveillance” in order to trigger an early response, have been proposed [3]. However, our earlier literature review found that there were no systematic analyses or validations of these putative indicators or of their operational reliability and cost-effectiveness [4]. The published evidence-base is equally poor when it comes to defining what constitutes an effective and efficient outbreak response: remarkably, there are no proven methods recommendable for epidemic vector control [5,6] or for clinical-health systems management when there is a surge in cases. In a systematic literature review about the few documented experiences with outbreak response some criteria were highlighted [7] in relation to a) management of outbreak response (multidisciplinary response teams, Incorporation of public organisations, written information for mass media, monitoring and evaluation of all control activities), b) management of vector control (‘search and destroy’ teams, communities involvement, geographical coverage of activities, enhanced surveillance, education of households) and c) management of health services (staff training-including laboratory staff), mosquito nets in hospitals, establishing case report conferences, adequate supplies for laboratory and case management).

Following the efforts by Gubler et al. [8] to sketch the surveillance efforts in a number of dengue endemic countries, there was a first attempt to define best practices in dengue surveillance in Latin America and Asia through expert meetings [9]. This resulted in a number of useful general recommendations: dengue should be notifiable, regional disease classification applied, electronic reporting developed, laboratory networks initiated, reporting focussed at the essentials, additional studies done and early prediction of outbreaks achieved.

To the best of our knowledge however, comparative studies on dengue surveillance and outbreak response using the same methodologies across participant countries have not yet been published. To get a more comprehensive view of existing surveillance systems and early outbreak detection ability, we analysed the contingency plans from 11 countries, updated the systematic literature review on dengue surveillance (publications in preparation) and report here on the analysis of dengue surveillance, outbreak detection and response in10 countries in Asia and Latin America. The purpose is to contribute to the development of a new evidence-based model for dengue surveillance and outbreak response, adaptable to individual country requirements and capacities, combining the best of existing strategies with a framework for the acquisition of evidence for novel approaches and tools. On the basis of the findings presented in this paper a model contingency plan will be developed, adapted to country needs and prospectively tested in different settings.

## Methods

This study used a mixed methods approach combining key informant interviews, document analysis, focus group discussions, secondary data analyses and interpretation by an international dengue expert meeting. In order to get representative information on dengue endemic countries, five countries in Latin America (Brazil, Colombia, Dominican Republic, Mexico, Peru) and five countries in Asia (Indonesia, Malaysia, Maldives, Sri Lanka, Vietnam) were selected, based on the following criteria: a) representation of large (e.g. Brazil, Indonesia), intermediate (e.g. Peru, Vietnam) and small countries (e.g. Dominican Republic, Maldives) in each region; b) recent dengue outbreaks; c) existing relations with Ministries of Health and academic institutions to ensure openness and confidence between respondents and interviewers. Three countries were excluded as they had been analysed previously in a WHO-TDR supported study: Thailand, Cambodia and Bolivia (Runge-Ranzinger et al. unpublished data). Within the study countries, academic institutions and/or Ministries of Health were approached to select country interviewers who had public health knowledge, understanding of both disease surveillance and dengue, and skills in undertaking interviews. The interviewers received detailed instructions and data collection forms that roughly followed the Centers for Disease and Prevention “framework for evaluating public health surveillance systems for early detection of outbreaks” [3] and had been pre-tested and revised following use in 3 countries (Nepal, Bangladesh, and Colombia). The set of data collection forms comprised:

a) Questionnaire for interviewers about the country context (data to be extracted from published and unpublished documents in the country).

b) Questionnaire/topic guide “Evaluating dengue surveillance and response” that included 83 items to be covered in relation to dengue disease surveillance, case notification and dengue classification, virological surveillance, routine vector surveillance and control, community participation during non-epidemic periods, outbreak preparedness, outbreak detection, recent experiences with a dengue outbreak and opinions about the success of the outbreak response.

c) Hospital questionnaire with 38 items about outbreak preparedness in hospitals, available resources in non-epidemic periods, characteristics of the last dengue outbreak, availability of resources during the outbreak, opinions about successes and failures of outbreak management.

d) Topic guide for focus group discussions to be applied in hospitals with a recent dengue outbreak

e) Matrix for the summarisation of collected information.

The work within countries included the following:

1. General appraisal of the epidemiology of dengue in the study country (questionnaire for interviewers mainly to be filled at national and State/Provincial level).

2. Semi-structured interviews using a list of 83 topics to be discussed with the interviewees (questionnaire/topic guide “evaluating dengue surveillance and response”). These data collection forms, developed with the above-mentioned framework for evaluating surveillance systems in mind (Buehler et al. 2004), were applied to key informants in each country at different levels: a) Government officials (central level): decision or policy maker, epidemiologist/ surveillance expert, laboratory expert (microbiologist or technician), entomologist, other. b) Government officials (state/province/district, sub-district level): epidemiologist/surveillance expert, laboratory expert (microbiologist and/or laboratory technician), entomologist, other. The number of interviews to be conducted followed the “saturation principle” (when no further information could be collected from informants the interview series was terminated) and included in general 20 to 30 respondents per country.

3. The hospital questionnaire with 38 items was applied in each country in 3 to 5 hospitals of different levels (teaching hospital, district hospital, sub-district hospital) using a purposive sampling strategy. At most sites there were none or only a few more similar hospitals in the study area.

4. Focus group discussions [10] with hospital staff involved in the last dengue outbreak were conducted for complementing the information obtained from individual interviews.

Combining complementary methodologies and information from varied sources, as well as several rounds of data verification before, during and after the expert meeting ensured a high level of internal validity providing more reliable results.

Ethical approval was received from WHO Regional Offices (Ethical Review Boards at the Pan American Health Organisation (PAHO), South-East Asian Regional Office (SEARO) and the Western Pacific Regional Office (WPRO) which was accepted by the study countries except for Peru where an additional ethical approval was obtained from the Institutional Review Board at Cayetano Heredia University. Verbal, and in some cases, written consent was obtained from respondents. The interviewees were assured of the anonymisation of their responses and interviews took place in closed rooms with no other persons being present. The completed forms were kept in separate files and no individual names of the respondents were recorded.

The country interviewers completed the data collection in the following areas: Brazil: : Brasilia Ministry of Health (National level). Pernambuco, Amazonas, Rio de Janeiro and Goiás (State level). Interviews at health units and hospitals in Amazonas and Rio de Janeiro (local level).; Colombia: : Bogota (National Institute of Health National level), Departamento Valle del Cauca (State level).; Dominican Republic: : Santo Domingo (National level) Santiago Valverde, San Juan, Azua, and Hato Mayor (Provincial level), Laguna Salada, Esperanza y Cienfuegos (Municipality level).; Mexico: : Mexico City Ministry of Health (National level), Yukatan and Guerrero (State level).; Peru: : Lima Ministry of Health (National level), Loreto and San Martin (State or Regional level), interviews in hospitals of Iquitos (Loreto), Tarapoto and Moyobamba (San Martin local level).; Indonesia: : Jakarta (National Provincial and District level) Yogyakarta (Provincial level) Bantul (District level).; Malaysia: : Kuala Lumpur Ministry of Health, Federal Territory of Putrajaya (National level), Selangor, Penang (State level) Klang Hulu Langat, Gombak , Petaling, Banting, Kuala Selangor(District level).; Maldives: : Male Ministry of Health (National. level), Hulumale, Thinadoo, Addu (Regional and District level).; Sri Lanka: : Colombo Ministry of Health, Medical Research Institute (National level), Colombo and Gampaha district (District level), hospital interviews in three hospitals in Colombo and Gampaha District (local level).; Vietnam: : Ha Noi Ministry of Health and National Hospital of Infectious Diseases (National level) Ho Chi Minh City (Hospital for Tropical Diseases District 8 hospital, Pasteur Institute, University of Medicine and Pharmacy, Preventive Medicine Centre of Ho Chi Minh City) Dong Thap province (Provincial Hospital Dong Thap Preventive Medicine Centre and Volunteer Group for Dengue). 

The field work was done within a 5 month period (October 2011 to March 2012). The interviewers produced a comprehensive report which included a detailed analysis of the dengue epidemiology in their country, the completed data collection forms and the completed matrix with the summary of findings of each item in the questionnaire/topic guide “Evaluating dengue surveillance and response”. The information package was sent to the central team at WHO-TDR for compilation and preliminary comparative analysis. A 24 page synopsis of findings for both Latin American and Asian countries was produced. It was circulated among interviewers, Ministry of Health staff in the participating countries and WHO focal points for verification and complementation. Thereafter in June 2012 a three-day international expert workshop was organised by WHO-TDR involving all interviewers and two representatives from Ministries of Health of each country. The 45 participants were tasked to a) further validate the collected information; b) interpret and regionalise country findings and c) discuss recommendations. Expert consensus was gained through mediation by the Chairpersons. The following section provides the results of a synoptic analysis of the 10 study countries.

## Results

Comparative results of the 10 study countries included the description of the surveillance system (purpose, stakeholders and operation –based on interviews and document analysis), mechanisms of outbreak detection (timeliness, validity, validation approaches, quality assurance of data –based on interviews), country experiences with the last outbreak (based on interviews, and document analysis) and expert opinions about prospects and limitations (based on interviews) [3]. The information was summarised in the above mentioned data matrix and further analysed during the expert meeting. The presentation of results in the text below follows a more functional order. An overview of findings is presented in Table 1.

**Table 1:** Overview of findings on dengue disease surveillance, vector control, epidemic preparedness and outbreak response

**Organisation of disease surveillance**	
National guidelines, obligatory notification	All study countries had guidelines for surveillance and obligatory notification of suspected and confirmed cases of dengue (in Asia only the severe forms of the disease). No private sector reporting except Brazil and parts of Vietnam
Laboratory confirmation	All Latin American countries attempted 100% confirmation (IgM/IgG); in Asia < 10%
Data transmission	Mainly electronic: Brazil, , Colombia, Dominican Republic, Mexico, Malaysia, Maldives, Sri Lanka (partly) Mainly paper based, partly electronic: Peru, Indonesia, Vietnam, Sri Lanka
Data analysis	At sub-national (state, department, province) and national level. Dominican Republic mainly at national level.
Case classification	Revised WHO classification [5] for clinical management throughout Latin America, Indonesia (partly), Malaysia (partly), Vietnam the others used the dengue fever/dengue haemorrhagic fever/dengue shock syndrome classification.
Active surveillance in sentinel sites	All countries (Maldives only during outbreaks)
Use of alert signals	Most countries collected information on different signals without using it for response (see below)
Routine evaluation of the surveillance system	Weak in all countries except for Brazil, Colombia and Sri Lanka
**Organisation of vector surveillance including community involvement**	
Larval surveys and outbreak alert	Conducted in all countries (Maldives and Vietnam only in sentinel sites). Some indices used for outbreak alert (Peru, Indonesia, Sri Lanka,)
Routine vector control	Larviciding with temephos or Bti (all countries except Indonesia, Maldives, Vietnam)
Vector control issues	Lack of resources, supervision and local involvement in decision making. Vector resistance. Difficult interpretation of entomological indices.
Social mobilisation	All countries use IEC materials, some use the COMBI method
**Epidemic preparedness and outbreak response**	
Outbreak response plans	All countries (except Maldives, Sri Lanka) with varying quality and details
Outbreak response committee	Defined in all countries (except Maldives)
Outbreak definition	Variable definition across countries and in some cases within countries. Some countries with no clear distinction between outbreak and seasonal peak. Several countries use the 2SD of weekly cases above the historical mean or the “moving average” (see text)
Delay of outbreak response	Difficult to assess in most countries due to lack of outbreak definition and delayed reporting. Time lag seems to be usually above 2 weeks but often much longer.
Alert signals and early response	Signals used: entomological indices, increased virus positivity rate, change of serotype, increased case numbers, increased number of fever cases, increased population movement. Information on several signals is collected in some countries but not used for early response because of uncertainty about the validity of the trigger (particularly entomological indices), budget limitations, staff shortage and delay in analysis.
Successful response activities	To a certain extent satisfactory vector control (all countries), improved clinical management, improved coordination (intra-and inter-sectoral) and better information systems (selected countries)
Room for improvement	Improved planning, training, involvement of local staff, enhanced community participation, faster solution of budget constraints, and better cooperation among neighbouring municipalities.
Coping with dengue outbreaks in hospitals (surge capacity)	Positive experiences: Epidemic response plans (in about half of study hospitals); establishing special dengue treatment units; stock-out management (in a few hospitals). Issues were: getting additional beds and staff, timely resource allocation, stock-out management (particularly intravenous fluids and blood products) and clinical management by untrained staff.

### Organisation of dengue disease surveillance

#### Characteristics of the surveillance systems and reporting

All five Latin American and five Asian countries had national guidelines for disease surveillance, some with detailed instructions, others in a very general form. All countries had a public health surveillance system that included a large number of infectious diseases, but some countries had at national level a special dengue unit (Brazil, Dominican Republic, Indonesia, Sri Lanka and Vietnam). Dengue notification was mandatory in the study countries- in Asian countries only hospitalised cases or Dengue Haemorrhagic Fever cases- with the exception of the Maldives. Reporting by the private sector was irregular or almost non-existent with the exception of Brazil and parts of Vietnam. In all countries both suspected and confirmed dengue cases were reported. All Latin American countries and some Asian countries (Vietnam, partly Malaysia and Indonesia) used the revised WHO dengue case classification (dengue/severe dengue [5]) for clinical case management (in Brazil with modifications) and for reporting. However, the surveillance system in Brazil and Mexico at the time of the study used the dengue fever/dengue haemorrhagic fever/dengue shock syndrome (DF/DHF/DSS) system which was perceived by some respondents to be a problem. The DF/DHF/DSS system was also used in parts of Malaysia, the Maldives, Sri Lanka and Vietnam.

#### Time-lag for reporting, data transmission and analysis

The time-lag for the reporting of suspected cases to the central surveillance system was, according to the respondents, only 1 to 3 days either from onset of symptoms or discharge from hospital (according to reporting rules in each country) with the exception of holiday periods or major festivities when the information flow was interrupted and outbreaks remained undetected (Colombia and Peru). No sound analysis of the delay in reporting could be found except for Brazil where more than 90% of cases were reported within 10 days. The mode of data transmission within the surveillance system was mainly electronic in Brazil, Dominican Republic, most parts of Mexico and Colombia, Malaysia (“e-dengue”), Maldives and parts of Sri Lanka and Vietnam. Paper forms, usually transmitted by fax or sometimes read out over the telephone, were used in remote areas of the Dominican Republic, most parts of Peru, Indonesia, Vietnam (partially electronic) and Sri Lanka. In Latin America severe cases were reported daily (in Brazil immediately) and suspected cases weekly. Also in Malaysia and Vietnam daily reporting was the rule and in other Asian countries weekly reporting. The analysis and aggregation of data was mainly done at regional level (state, department, region) and then sent to the national level. In the Dominican Republic the analysis was mainly performed at the national level. Peri-focal interventions (if any- see below) were performed at local level using data from the case reporting form. Geocoding of surveillance data was not usually performed.

#### Laboratory confirmation

All Latin American countries attempted to carry out laboratory confirmation of all dengue suspected cases, mainly with IgM and IgG ELISA and increasingly with NS-1 However, during outbreaks a small fraction of suspected cases were tested: at least 10% in Brazil and 30% in Mexico. In contrast, the laboratory confirmation of dengue in Asian countries–generally using IgM/IgG ELISA- was done on a small sample of patients (usually less than 10% of patients), though laboratories used the same tests as Latin America. The delay of serological confirmation was said to be usually 3 days within large hospitals; in Sri Lanka virus isolation took 2 weeks or longer.

#### Active (enhanced) surveillance

Active surveillance-particularly virus surveillance- in sentinel sites (mainly hospitals) was practiced in all countries, but in the Maldives only during outbreaks. Viral surveillance including serotyping and genotyping was usually done by national laboratories except in Maldives and Dominican Republic where it was done in neighbouring countries. DENV 1 to 4 was circulating in all study countries except the Maldives where the situation is unknown. The main purpose of viral surveillance was to detect the introduction or shift of sero/genotypes for describing viral trends. However, this information was only used as an alert signal for a dengue outbreak in Malaysia and sometimes in Vietnam and Brazil, but no concrete response activities were linked to it (see section on alert signals).

#### Monitoring and evaluation of the surveillance system

The surveillance system itself had been evaluated systematically in some Latin American countries (Brazil and Colombia, partially in the Dominican Republic) but sero-surveys had not been used to establish sensitivity/specificity of dengue reporting or to calculate a correction factor. Within Asia, particularly Sri Lanka has an established monitoring and evaluation (M&E) mechanism for its surveillance system; in the other countries M&E activities were restricted to the occasional assessment of data quality or to local projects (Malaysia, Maldives). Serological surveys were in use in Malaysia and Vietnam for assessing trends but not for estimating under or over-reporting of dengue cases. Routine attempts to estimate the level of over- and under-diagnosis of dengue were not in place in most countries.

#### Staff training

Epidemiologists working on disease surveillance had had some basic training (except for Peru and Mexico) but there was generally no “on-the-job-training” in place. Only Malaysia, Sri Lanka and occasionally Indonesia offered refresher courses. High staff turn-over was a problem in some places (particularly mentioned in Brazil, Colombia and Maldives). The general view was that there was sufficient expertise at national and regional level –with considerable variation particularly within the large countries- but not so at operational level.

### Organisation of dengue vector surveillance

Vector surveillance by larval surveys was done routinely in all countries by vector control staff (in Vietnam only in “sentinel communes”, in Maldives only in some municipalities and in Brazil country-wide using a well-established rapid system called LIRA). Pupal surveys for determining productive container types for targeted interventions [11] were occasionally carried out in some countries; in others it was not clear how the identification of productive containers was done. In all countries the standard larval indices (HI, CI, BI) were used but specific indicators were used for outbreak alert (BI in Sri Lanka, HI and BI in Vietnam and Indonesia, HI in Peru). In some countries the vector control staff have the legal power to enter premises (Dominican Republic, Colombia, Malaysia, Sri Lanka, Indonesia during outbreaks). Routine vector control as a year-round practice was mainly done with 1% temephos WG granulate (all countries except for Indonesia, Maldives and Vietnam) and in some countries with *Bacillus thuringiensis*, Bti (Brazil, Colombia, Maldives). Biological control was implemented in some limited parts of Vietnam (copepods) and larvivorous fish were used in Sri Lanka. Source reduction was strong in big cities of Indonesia (“3-M campaign”, clean, cover, remove water containers). Issues of routine vector surveillance and control as identified by respondents were: a) Lack of resources (Colombia, Mexico, Indonesia, Maldives, Sri Lanka, Vietnam); b) Lack of involvement of local level in decision making (Dominican Republic, Peru, Vietnam); c) Limitations in supervision and increasing vector resistance to larvicides (Brazil); d) Difficulty in interpretation of entomological indices (Malaysia, Sri Lanka).

### Organisation of community participation

Social mobilisation programmes were present in all countries and territories to varying degrees. Methods such as COMBI (communication for behavioural impact, mentioned in Dominican Republic, Malaysia, Maldives and Indonesia) were popular. Information-education-communication material (IEC) was also used with other approaches: house visits, dissemination of flyers, didactic materials in schools, banners and posters. The messages were mainly delivered by health staff and/or volunteers and religious leaders. The main barriers to a successful programme were perceived to be budget limitations, lack of a continuous and systematic approach with no impact evaluation and often no empowerment of communities.

### Epidemic preparedness and outbreak response

#### Response plans

All countries (except Maldives; Sri Lanka plan not available at the time of the study) had an outbreak response plan, some with detailed instructions, others in very general terms (see analysis of contingency plans). In some countries these plans had been made widely available; in others they were mainly known and used at national level. Some plans included instructions on training but this was rarely implemented. Outbreak response committees were defined in all countries (except Maldives); they comprised major stakeholders from the public sector under the direction of the Ministry of Health (MoH); in a number of countries these also comprised stakeholders from other Ministries and Agencies (Brazil, Mexico, Malaysia, Sri Lanka, Vietnam).

#### What constitutes an outbreak?

Definitions of a dengue outbreak showed marked differences and in a number of countries several, often incompatible definitions were in use. Only in 6 out of 10 countries was a fairly consistent definition applied:

a) Case numbers 2 Standard Deviations (SD) above the mean of the preceding five years shown in endemic channels (Colombia, Dominican Republic, Peru (partially), Vietnam -national level). In Brazil and Malaysia the “moving average” (mean or median) was used, i.e. the “4-weekly average” above the mean of “three 4-weekly averages” in the five preceding years.

b) >300 cases per 100,000 population at local level (Brazil); > 10 cases per week in a local area (Sri Lanka). Case number within a “commune” within 2 weeks: 2–20 cases = mild outbreak; 20–100 cases=moderate outbreak; >100 cases = severe outbreak (Vietnam).

c) Two or more connected dengue cases at local level (Mexico, Malaysia, Sri Lanka partially).

d) Continuous increase of dengue cases for 2 periods (hours, days, weeks) or double the number of cases within a month compared to the previous year, or 50% increase in case fatality rate, (Indonesia).

e) No outbreak definition (Maldives); no clear outbreak definition but larval indices as trigger for response: BI <6= routine response; BI= 5-20= house-to-house checks; BI> 20 = fogging (Sri Lanka).

#### Characteristics of dengue outbreaks

Due to country variation in outbreak definitions it was impossible to compare a number of variables, including the duration of a dengue outbreak, total number of cases, cases during the worst week, and geographical distribution. This applies particularly to those countries that did not distinguish between seasonal increase of cases and outbreak; only the number of cases at the end of the wet season (“dengue season”) could be estimated. In countries with an outbreak definition the average duration of a dengue outbreak at provincial, regional and state level was recorded as 10.2 months (range 5 to 13 months) and the average number of cases was 26 732 (range 12 171 to 69680 cases). In Brazil the average incidence rate of dengue cases during outbreaks was 538 per 100 000 population, clearly in excess of the 300-threshold (detailed analysis of a set of dengue outbreaks to be published by the Brazilian partner).

#### Time-lag between outbreak detection and response

Again, due to the lack of a standardised outbreak definition it was difficult to establish the time-lag between start of the outbreak and response, but in some countries the following information was collected:

a) Two to four week gap from the start of a sudden increase in cases to the interpretation of information for decision making (Brazil; to be confirmed by an on-going analysis of Brazilian dengue outbreaks).

b) Fast recognition of the start of an outbreak (2 to 5 days) but delay for several weeks to get the response in place (Dominican Republic). Similar situation in Mexico where the delayed allocation of extra budget was an issue.

c) Slow interpretation of case increase (1 month) and 2 additional weeks for start of the response (Peru, Maldives).

d) Confusion about the start of the outbreak but then a relatively fast response (1 week) in Mexico.

#### Alert signals (risk indicators) for outbreaks

Alert signals for a dengue outbreak were used differently and often un-systematically. These include:

a) Increase in cases > 2SD above the mean of previous years (Colombia, Dominican Republic, Mexico, Peru with different thresholds defined by individual states) or above the “moving average”(Brazil, Malaysia–see outbreak definition).

b) Elevated entomological indices: Breteau Index (BI) and/or House Index (HI) (Peru and Vietnam), BI (Sri Lanka), BI/HI/Container Index determined by rapid routine survey LIRA (Brazil).

c) Increased virus positivity rate in blood samples (Dominican Republic).

d) 25% more cases per week compared to previous year (Dominican Republic) or 50% increase (Indonesia, Bantul and Yogyakarta).

e) Change of serotype (Peru) or increase in dengue incidence during dry season (Peru).

f) Various other signals were monitored by surveillance systems but they were not linked to early responses: increased rain fall and temperature (in all study countries), recent displacement of dengue patients into non-endemic areas (Colombia) and population movements (Sri Lanka).

Thus in some countries the “alert signals for an outbreak” are the same as the “outbreak definition” leading to late alert and delayed response. The following issues mentioned by the respondents hampered the prediction/early detection of a dengue outbreak:

a) Low capacity of clinical and entomological alert signals to predict an outbreak (Brazil). Restricted number of variables included in the list of alert signals (Colombia, Mexico). Analysis of alert signals only on a monthly basis (Indonesia).

b) Inadequate preparation of municipalities and states in the use of alert signals (Brazil). Shortage of staff and training opportunities (Sri Lanka). No strict application of alert signals at sub-national level (Dominican Republic, Mexico).

c) Budget limitations during inter-epidemic periods (Colombia, Mexico) and during response activities (Vietnam, Indonesia).

d) No outbreak declarations issued (Vietnam) or issued very late (Maldives).

#### Outbreak response

In all study countries the response to a dengue outbreak was carried out first by the vector control services and included outdoor space spraying, and in some countries indoor fogging in critical areas. In all countries, additional clean up campaigns, larviciding with 1% temephos WG and exceptionally with Bti was used. These activities were often complemented by larval surveillance. Community involvement was reported to be strong in Indonesia and parts of Maldives.

Country respondents mentioned a number of successful activities in dengue outbreak response. These included successful vector control interventions (all countries), improved clinical management (due to better clinical training and obligatory analysis of dengue deaths); leadership and better inter-sectoral cooperation in outbreak response (Brazil, Peru, Malaysia, Sri Lanka, Maldives, Indonesia), improved information system (Brazil, Sri Lanka).

Room for improvement of outbreak response, additionally to the request for a standardised outbreak definition, was mentioned including:

a) Earlier outbreak detection, planning, administrative procedures (Dominican Republic, Peru, Sri Lanka) as well as improved budget and/or budget planning (Colombia, Peru, Maldives, Sri Lanka) and cooperation among municipalities (Brazil, Dominican Republic) with locally developed plans (Mexico).

b) Better training (Colombia, Brazil, Peru, Indonesia, Sri Lanka) particularly of local health staff and civil servants (Brazil, Dominican Republic, Maldives).

c) Better community involvement and promotional activities (Colombia, Mexico, Indonesia, Malaysia, Sri Lanka,) including improved routine vector control (Colombia, Mexico, Indonesia, Malaysia, Maldives, Sri Lanka).

### Surge capacity (coping capacity) of hospitals with dengue outbreaks

In the five Latin American countries 21 hospitals were analysed. Six hospitals were small (20–30 beds, Dominican Republic only), 13 hospitals were middle sized (90 to 400 beds) and 2 were large (above 400 beds, Colombia and Dominican Republic). The annual bed occupancy rate was 70% to (exceptionally) 100%.

In the five Asian countries 20 hospitals were analysed. Two were small hospitals (50 to 100 beds), 6 were middle sized (100 to 400 beds) and 12 were large (more than 400 beds). The annual bed occupancy rate was 80% to 120%.

The last dengue outbreak in the study countries had occurred 18 months to 4 months preceding the interviews. Epidemic response plans existed in 9 out of 21 Latin American hospitals; most of these plans included recommendations for dealing with stock-outs. In Asia 12 of the 20 hospitals had epidemic response plans and particularly Malaysia, Sri Lanka, Vietnam had special dengue units for enhanced dengue clinical management.

During the dengue outbreak operational problems occurred in most hospitals and different coping strategies were applied. Special dengue units were perceived to be very helpful for clinical management (particularly in Peru, Malaysia, Sri Lanka and Vietnam).

Additional beds were made available in 13 of the 21 Latin American Hospitals, using mainly stretchers, discarded beds or beds from other wards. Asian hospitals used trolleys and foldable beds (Malaysia), mattresses on the floor (Sri Lanka) or two patients in one bed (Vietnam). Sometimes dengue patients had to be discharged earlier, elective surgeries had to be cancelled or in exceptional cases non-dengue patients had to be referred to neighbouring hospitals. A strategy adopted by Brazil to reinforce the hospital capacity was to establish tents with beds for intravenous fluid and observation.

Additional staff could be hired in 7 Latin American hospitals but extended shifts and transfer of staff from non-dengue wards was the usual strategy. Dengue patients were discharged early or referred to another hospital in just 2 hospitals. However, no negative implications for non-dengue patients were reported. In Asia, there was transfer of staff from non-dengue wards when necessary (Malaysia) or staff had additional or prolonged shifts (all other countries). In some instances relatives helped with monitoring fluid balance. Stock outs, mainly intravenous fluids- occurred in at least 5 of the 21 Latin American hospitals and shortage of reagents was reported in 4 hospitals. Brazil had a positive experience with a recently established laboratory network which was able to handle the surge in specimens. In 3 Asian hospitals stock-outs of colloid solutions and blood products were reported; in one hospital laboratory tests had to be outsourced to private clinics.

## Discussion

### Prospects and limitations of the study

This study details a novel attempt to use a standard methodology across 10 Latin American and Asian nations to characterise country experiences of dengue surveillance, outbreak detection and response. As with all qualitative data, subjective bias may have been introduced through the opinions and perceptions of individual respondents; variability in responses was noted between country interviewees in the following areas: reporting delays, timeliness of outbreak detection and response and assessment of effectiveness of interventions. However, the use of complementary data collection methodologies, combined with several rounds of verification with country representatives at the WHO-TDR expert meeting ensured the triangulation of information collected from various sources and facilitated a high level of internal validity. Whilst any attempt to fully characterise the global situation of dengue surveillance and outbreak detection/response would require a stratified random sampling of countries, the detailed data gathered and presented here from a diversity of countries in both Latin America and Asia provides useful information towards the development of an evidence-based model contingency plan for dengue outbreaks.

Based on the data presented in the previous section, the WHO-TDR expert meeting with country representatives identified best practices, reasons for failures and research needs. The discussion below focuses on key areas identified for improvement.

Need for distinguishing between “expected increase in cases” (i.e. seasonal peak) and “unexpected increase in cases” (i.e. an outbreak).

**Figure 1:**
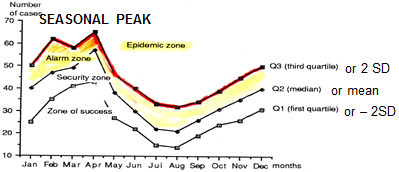
**Illustration of the seasonal variation of a vector borne disease like dengue.** An example of an ‘endemic channel’ is shown here. The ’expected increase in cases’ usually coincides with, or follows, the rainy season. The shaded area corresponds to an ‘alarm zone’ where case numbers reach levels above the mean (or median) of a preceding time period (for example 5 years). The ‘epidemic zone’ is entered when case numbers reach levels above 2 standard deviations (or the third quartile).

Our findings show that most countries did not distinguish between a seasonal rise in dengue cases, usually during the rainy season (see Figure 1), and the unexpected increase in cases above a defined threshold, usually called an outbreak [2,12]; the number of reported cases exceeding expected levels is referred to as “aberrations” [13]. The need for dengue control and clinical care systems to respond differently to each of these scenarios was identified. The expected increase of dengue vectors and subsequently of cases during the “dengue season” requires routine measures be stepped up at a relatively predicable point each year. The annual need for increased vector control staff should correspond to the weeks when the vector density increases and preparations should be made for adequate staffing levels, equipment and supply (including chemicals and/or biological agents, IEC materials and other elements of social mobilisation). Likewise, clinical services should define in their annual plans the additional staff, equipment, reagents and treatment units needed and whether clinical refresher courses are required. The dengue outbreak as an “unexpected increase of cases” requires additional efforts that are described below.

### Need for an agreed outbreak definition

The data on country experiences shows a wide range of definitions used for defining dengue outbreaks, sometimes leading to confusion for stakeholders and delayed emergency outbreak responses. Discussions at the expert meeting identified the importance of a generally agreed outbreak definition. Many countries use a version of the “endemic channel” for visualising the expected case levels, based on the weekly (or monthly) average number of cases over the preceding 5 years. Above this is a line that represents +2SD; others use the median and the 3^rd^ quartile (Figure 1).

**Figure 2:**
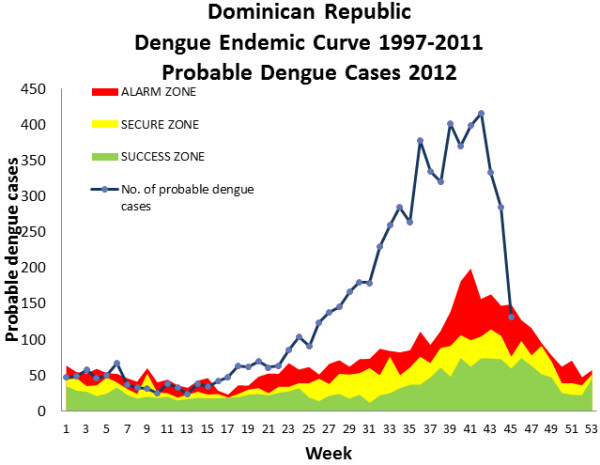
**An example of an outbreak curve of case numbers from the Dominican Republic is shown here.** The number of new cases crosses the “historical” +2SD line from week 1 to 17 several times before, in week 18, the case numbers definitively rise.

**Figure 3:**
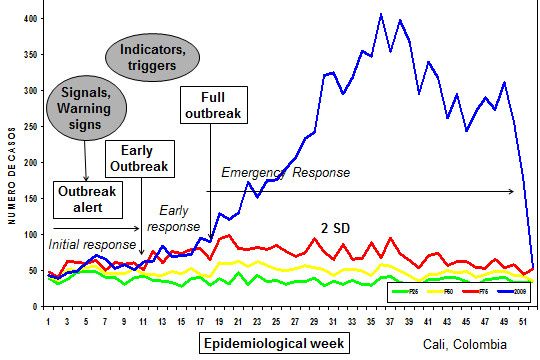
**Illustration of the different phases of a dengue outbreak and different levels of response.** An example of an outbreak curve from Colombia in 2009 is shown here. An ‘outbreak alert’ is identified early through a combination of ‘alert signals’, and triggers an ‘initial response’. The evolution into ‘early’ and ‘full outbreaks’ are detected early using standard definitions and trigger appropriately staged ‘early’ and ‘emergency responses’.

The area between the lines of the mean and +2SD is called “alert zone” or “alarm zone” and the area above the +2SD line or Q3 line is called the “epidemic zone” showing the aberrations (Figure 2). If the weekly number of dengue cases crosses the “historical” 2SD line, then it is called an “outbreak”. Figure 3 shows a dengue outbreak with the blue line of “weekly number of cases” crossing the +2SD line several times between week one and 17 until the case numbers shoot up in week 18, a typical pattern observed in dengue outbreaks (Siqueira, unpublished data).

According to the WHO-TDR expert meeting the following *advantages* of the “+ 2SD” outbreak definition were identified: a) The “endemic channel” is a simple instrument and the crossing of the 2SD line is easy to assess; b) a standard definition of a dengue outbreak can be used to inform the mass media and the public about the actual situation; c) it is possible to determine the size of an outbreak in terms of duration, total number of dengue cases, case fatality rate and thereby facilitate in-country and cross-country comparisons; d) it helps to assess the effect of response mechanisms and to define “stopping rules” (when the intensified response can be terminated).

Possible *disadvantages* of such a definition were also identified: a) Programme managers may be tempted to use the crossing of the +2SD line as a “warning sign” for an outbreak (rather than as an indicator that an outbreak is effectively already underway) and initiate a delayed emergency response; b) the definition has limited sensitivity (only 40% of such events when case numbers crossed the +2SD threshold were followed by a “massive” increase of cases in Puerto Rico [13]; using a similar predictor with a 1 SD threshold, Barbazan et al. [14] found a sensitivity of 66%) c) outbreaks in previous years can result in thresholds that are too high; no satisfactory algorithm for recognising past aberrations has been devised [12]; d) the seasonal increase in cases may come earlier than in the 5 preceding years providing the impression of an outbreak. This phenomenon has been handled by using the “deviation bar chart” [13] or the “moving average” applied in Brazil and Malaysia (“moving median”). In this case, the average of dengue cases during 4 weeks is compared with the average of cases during a period of 12 weeks in the preceding 5 years (i.e. the same 4 weeks as the actual observation period plus 4 weeks before and 4 weeks after the observation period [15]). In Puerto Rico the sensitivity of such a “deviation bar chart” for indicating a dengue outbreak was 40% and the specificity was 90% [13] e) when the geographical units are too small, the variation of cases increases and may show wide oscillations.

Other outbreak definitions, for instance the” incidence threshold” (i.e. when the number of cases during a week passes a pre-defined threshold level, such as 300 per 100,000 population as used in Brazil) need more research regarding sensitivity and specificity. The definition of “two interconnected dengue cases” should be limited to an outbreak definition in non-endemic areas; however, it may be used to trigger routine operations (peri-focal interventions) in local areas.

It was felt by the expert meeting that a clear and universally accepted definition of an outbreak is important and that the advantages outweigh the disadvantages. A standardised outbreak definition can help to send uniform messages to inform the general public and make the outbreak analysis comparable within and between countries. However, it has to be emphasised that the response to an outbreak should start much earlier and not as an “emergency response” (see below).

### Need for identifying alert signals (warning signs, risk factors, response triggers)

Control measures come probably too late when they are implemented at the start of an outbreak (“late or emergency response” [16]). It is therefore recommended to react in a timely and structured way to an algorithm of alert signals for a dengue outbreak. The combination of these signals may vary between countries and also depend on the availability of resources. For example, some countries routinely perform virological testing on suspected dengue blood samples and use an increase as a warning sign (as in the Dominican Republic); however no standardised response to the increase of viral positivity is defined. As Farrington and Andrews [12] stated: “The real difficulty lies in…setting up appropriate protocols for deciding which signals to investigate and which to ignore and for communicating effectively the role and limitations of automated systems”. Some countries such as Brazil are testing a number of signals for use as outbreak predictors. Others collect information on climatic, viral and other variables but do not use them for outbreak alert. The use of BI or other larval indices with defined thresholds for different levels of response (see Sri Lanka), was controversially discussed as there is no evidence that larval indices reflect adult vector densities and the thresholds were felt to be arbitrary. Pupal indices seem to be more promising but need to be evaluated further [17]. It was emphasised that research on entomological alert signals, including the time lapse between increase of entomological signals and increase of case numbers, is urgently needed.

The expert meeting proposed a set of indicators (that will need to be tested and validated) as part of a scoring system that allows the definition of thresholds for epidemic responses (Table 2). Additionally an algorithm was proposed for further testing:

**Table 2:** Proposed alert signals (triggers for early response) as suggested by the WHO-TDR expert meeting (June 2012)

**Trigger**	Evidence or expert opinion**	**Feasibility**	Further research needed***
**New predominant serotype introduced**	+++	Most countries	+++
**Changes in age group distribution**	++	Most countries	+++
Increased number of hospitalised/outpatient fever cases/probable dengue*	+++	Most countries	+++
**Increase in vector presence**	++	Most countries	+++
**Increase in news reporting dengue outbreaks, social network comments**	+	Few countries	++++
**Climate changes: increase in rainfall/temperature/humidity**	++	Most countries	+++
Increase in % positive serology*	++++	Most countries	+
**Increase internal displacement/population mobility**	+	Context dependent	++++
**Cluster identified through GIS mapping**	++	Few countries	++
Identification of outbreak in a neighboring geographical unit (state, district, province, country)*	++++	Most countries	++

*Particularly useful indicators/ triggers.

** ++++ very strong; +++ strong; ++fairly strong; + weak.

*** ++++highest priority; +++ high priority; ++ necessary.

Early response will be triggered when any two of the following conditions are met at the local level in any given week:

1. Increase in dengue sero-positivity (level of increase to be defined).

2. Dengue outbreak in a neighbouring geographical area (district/province/region/country).

3. Increase of febrile cases /probable dengue cases in sentinel sites (level of increase to be defined).

In addition, the early response should be informed by entomological surveillance, geographical location of cases and shifts in the age distribution of dengue cases. The alert signals as triggers for response are presented in Table 2. They may vary from country to country as the surveillance systems collect different kinds of data.

### Research recommendations for outbreak detection and alert signals

The research needs presented in Table 2 were complemented by the following more detailed recommendations:

a) Retrospective and prospective analysis of different thresholds for outbreak detection (eg. 2 SD above mean; 1 SD; 50% above mean) and calculation of the sensitivity and specificity of these thresholds for subsequent outbreaks.

b) Long term representative sentinel based vector surveillance to assess the association of vector indices with clinical cases and the usefulness of alert signals based on virus surveillance, vector surveillance, climate data and others.

c) Prospective comparison of monthly incidence by province with virus surveillance data to see whether virus introduction/shift is followed by outbreaks (determine positive predictive value of such events) and with what approximate time lag.

### Need for different levels of outbreak response: initial response, early response, emergency response

Whilst our data showed that countries used varied and multi-faceted outbreak response mechanisms, the timing and intensity of these were often poorly informed and sometimes fragmented. In order to help initiate and direct staged response activities, the expert meeting identified the following stages of an outbreak (Figure 3):

1. Outbreak alert: when several alert signals point to a possible imminent dengue outbreak.

2. Early outbreak: when the weekly number of cases crosses the 2SD line in the endemic channel.

3. Full outbreak: when the case numbers increase rapidly.

After revising the country experiences, it was recognised at the expert meeting that each phase requires a tailored response:

**Initial response** during the alert phase includes: convening local dengue committees; activation of syndromic surveillance if not done on a routine basis; enhancement of routine activities (such as vector control and alerts for hospitals).

**Early response** during the early outbreak phase includes: convening local dengue or emergency operations committees; implementation of existing vector control guidelines; proper planning for contingency (human, financial and logistic resources) and intensification of focal intervention (vector reservoir control with community participation); social mobilisation (ICE, community participation, mass media partnerships); gathering of background information (cartography, demographics, inventory of facilities etc.); alerting hospitals and health centres (distribute guidelines for case detection and treatment; create or alert an outbreak management team); enhancement of surveillance and activation of syndromic surveillance and sentinel sites; enhancement of established communication channels (public health, clinical care, education system, media, the public, national and international authorities).

**Early response in clinical settings** includes: circulation of national guidelines on clinical management of dengue; staff training including officers in public and private hospitals; engagement of the private sector; establishment of dengue treatment areas in major hospitals and in high risk districts. Health services management should include: a) National/Provincial/District Steering Committees; b) the circulation of Hospital Dengue Preparedness and Contingency plans (which should include plans for mobilisation of doctors/nurses within the region and from other specialties, and plans for surge in bed requirement); c) health education messages for the community.

**Emergency response** (or late response) during the fully developed outbreak includes the full application of the contingency plans. The duration of the response measures needs to be defined. If these are terminated too early, the outbreak may simply be shifted to a later date but if the measures are stopped too late, resources may be wasted.

### Research recommendation for a staged outbreak response

The staged outbreak response suggested here will need to be tested prospectively for its feasibility, effectiveness in mitigating dengue outbreaks, cost-effectiveness (i.e. cost in relation to number of expected dengue cases avoided) and acceptance by all relevant stakeholders.

## Conclusions

**Figure 4:**
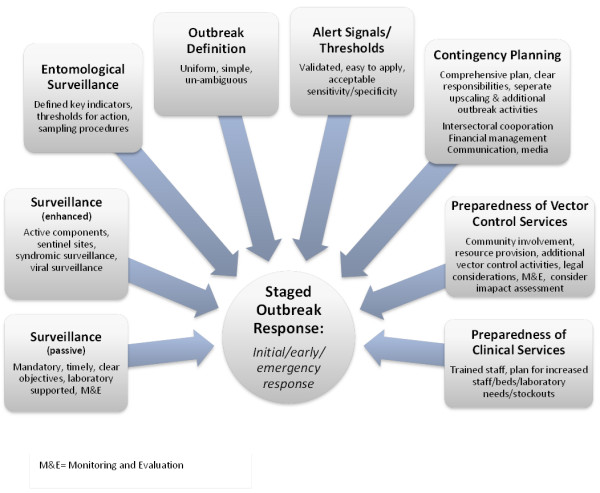
Essential elements of a surveillance and preparedness system to ensure an early, staged outbreak response.

The work presented here on lessons learned from country experiences in dengue surveillance, outbreak prediction, detection and response has demonstrated variation in practice and areas for improvement. Best practices were identified. They include *improved surveillance* (through electronic reporting, laboratory networks for mutual support and information exchange, standardised data collection, processing and feedback, strengthened monitoring and evaluation activities, enhanced capacity building) and improved *early outbreak detection* (through the use of pre-tested alarm signals and a standardised outbreak definition) in order to initiate a *staged outbreak response* (initial, early and late responses) applying predefined procedures. Further details are captured in Figure 4. There is increasing consensus that a certain level of standardisation, summarised in a model contingency plan, would help countries improve their preparedness for dengue outbreaks. This would also allow for greater cross-country sharing of experiences and ideas.

## Competing interests

The authors declare that they have no competing interests.

## Authors’ contributions

AK, SRR and JF designed the study. SB, DBV, EG, NK, HTT, PL, YM, RS, and AV collected the data. All authors were involved in data analysis, interpretation and writing the paper. All authors read and approved the final manuscript.

## Pre-publication history

The pre-publication history for this paper can be accessed here:

http://www.biomedcentral.com/1471-2458/13/607/prepub
